# Publisher’s Note: Continued Publication of *Tomography* by MDPI

**DOI:** 10.3390/tomography7010006

**Published:** 2021-02-09

**Authors:** Franck Vazquez

**Affiliations:** MDPI, St. Alban-Anlage 66, CH-4052 Basel, Switzerland; vazquez@mdpi.com

*Tomography* was launched in 2015 and has published international and multidisciplinary research on all aspects of imaging science, spanning from basic research to clinical trials. *Tomography* was published by Grapho Publications [1] over an initial 5 years and will be published by MDPI as of January 2021 [2]. Prof. Dr. Brian D. Ross [3], who launched this journal and has served as Editor-in-Chief since its inception, has kindly agreed to serve in this role for the next few months until a new Editor-in-Chief is appointed.

Alongside chemical and molecular probe advances, *Tomography* has published research about technical advances in hardware, software, and imaging informatics. *Tomography* is a unique publication venue that allows investigators to communicate integrated findings related to heterogeneous features associated with anatomical, physiological, functional, metabolic, and molecular genetic activities of normal and diseased tissue. *Tomography* will continue to publish papers involving content, space, and time-dependent analysis of multidimensional information. This will enable multivariate computational data extraction to identify select features of pathologic tissue phenotypes and advance imaging applications in basic science and clinical care.

We are delighted to take over the publication of *Tomography,* as it complements very well the portfolio of MDPI journals [4], provides a multidisciplinary addition to our existing journals in this area, such as *Journal of Imaging* [5] or *Radiation* [6], and strengthens our service to the relevant scientific communities.

MDPI will publish four quarterly issues as of 2021.

Looking forward to publishing your research work in *Tomography*!
